# Emerging roles of epithelial-mesenchymal transition in hematological malignancies

**DOI:** 10.1186/s12929-018-0440-6

**Published:** 2018-04-23

**Authors:** San-Chi Chen, Tsai-Tsen Liao, Muh-Hwa Yang

**Affiliations:** 1 Institute of Clinical Medicine, National Yang-Ming University, No. 155, Sec. 2, Li-Nong Street, Taipei, 11221 Taiwan; 2 Faculty of Medicine, National Yang-Ming University, Taipei, Taiwan; 3 Division of Medical Oncology, Department of Oncology, Taipei Veterans General Hospital, Taipei, Taiwan; 4 Cancer Progression Center of Excellence, National Yang-Ming University, Taipei, Taiwan; 5 Department of Otolaryngology, National Yang-Ming University, Taipei, Taiwan

**Keywords:** Epithelial-mesenchymal transition, Leukemia, Lymphoma

## Abstract

**Background:**

Epithelial-mesenchymal transition is an important process in embryonic development, fibrosis, and cancer metastasis. During the progression of epithelial cancer, activation of epithelial-mesenchymal transition is tightly associated with metastasis, stemness and drug resistance. However, the role of epithelial-mesenchymal transition in non-epithelial cancer is relatively unclear.

**Main body:**

Epithelial-mesenchymal transition transcription factors are critical in both myeloid and lymphoid development. Growing evidence indicates their roles in cancer cells to promote leukemia and lymphoma progression. The expression of epithelial-mesenchymal transition transcription factors can cause the differentiation of indolent type to the aggressive type of lymphoma. Their up-regulation confers cancer cells resistant to chemotherapy, tyrosine kinase inhibitors, and radiotherapy. Conversely, the down-regulation of epithelial-mesenchymal transition transcription factors, monoclonal antibodies, induce lymphoma cells apoptosis.

**Conclusions:**

Epithelial-mesenchymal transition transcription factors are potentially important prognostic or predictive factors and treatment targets for leukemia and lymphoma.

## Background

Epithelial-mesenchymal transition (EMT) is the process of epithelial cells transforming into mesenchymal cells. Cells that undergo EMT have typical morphological changes, including disruption of intercellular junctions, loss of cell polarity, reorganization of the cytoskeleton, and increased cell motility [1, 2]. Therefore, in most experimental models, epithelial markers (E-cadherin and α- and β-catenin), mesenchymal markers (N-cadherin, vimentin, and fibronectin) and morphological changes are examined as indicators to confirm the occurrence of EMT. EMT is classified into three types according to the EMT meeting held in 2007 [3]. These three types have different biological processes that lead to a distinct functional consequence. Type 1 EMT is associated with embryogenesis. At the stage of embryogenesis, EMT facilitates successful implantation [4] and gastrulation [1]. Type 2 EMT is associated with wound healing, tissue regeneration, and organ fibrosis. Type 3 EMT develops in epithelial cancer cells, which is associated with tumor invasion and metastasis, resulting in a worse prognosis.

EMT permits cancer cells to acquire migratory, invasive, and stem-like properties [5]. The reverse process of EMT, i.e., mesenchymal-epithelial transition (MET), is an important process for cancer cell re-differentiation and metastatic colonization [6]. The evidence linked between stemness and EMT is well documented. However, contradictory results have indicated the inhibition of EMT also promotes cancer stemness, which may be explained by the concept of partial EMT [7]. Furthermore, cancer stemness is independent of EMT, implying that EMT-TFs, the critical mediators for EMT, regulate cancer cells stemness separately from EMT in solid tumors [8].

Intriguingly, accumulating evidence supports the involvement of EMT-TFs in hematologic diseases. Twist1 is highly expressed in hematopoietic and leukemic stem cells [9]. The expression of Twist1 is implicated in hematopoiesis. Twist1 confers cancer cell self-renewal and apoptosis resistance both in solid tumors [10] and leukemia [11]. The Snail family also promotes lymphoid development by blocking apoptosis [12]. These findings have led to the investigation of the role of EMT-TFs in hematological malignancies. In this article, we will review the relevant studies on EMT-TFs in hematological malignancies and discuss the potential of EMT-TFs as biomarkers.

### EMT-transcription factors

One of the major triggering events for EMT is the activation of EMT-TFs, such as Snail1, Twist1, zinc finger E–box binding homeobox 1 (ZEB1), and ZEB2. These EMT-TFs often control the expression of each other and cooperate with other TFs to regulate the expression of target genes; EMT-TFs often function as repressors for epithelial genes and activators for mesenchymal genes [7, 13, 14]. The Twist family consists of Twist1 and Twist2, which are important EMT-TFs. Twist, a basic helix-loop-helix transcription factor originally recognized in Drosophila, is a master regulator of gastrulation and mesoderm development [15]. In humans, Twist is overexpressed in many types of cancers, such as head and neck cancer [16], hepatocellular carcinoma [17], and breast cancer [18], where it represents a poor prognosis. Twist1 induces EMT with the recruitment of methyltransferases to directly repress the promoter of *CDH1* that encodes E-cadherin [19]. Twist1 activates the transcription of *BMI1* and cooperates with it to enhance stemness [10]. Twist1 also suppresses let-7i to induce the mesenchymal-type movement of cancer cells [20]. Moreover, down-regulation of let-7i also activates the chromatin modifier AT-rich interacting domain 3B (ARID3B) to promote the expression of stemness genes through histone modifications [21]. To overcome oncogene-mediated senescence, Twist1 cooperates with Myc to override p53 and Rb-dependent programmed cell death [22]. In hypoxic microenvironments, hypoxia inducible factor-1 (HIF-1α) directly binds to the promoter of Twist1 to induce EMT [23]. Twist1 is also essential in TNF-α-induced EMT and is up-regulated by nuclear factor kappa-light-chain-enhancer of activated B cells (NF-κB) directly [24]. Twist2, which shares more than 90% identity with Twist1, also promotes EMT in human cancers. However, the expression pattern of Twist2 differs from that of Twist1 [25].

The Snail family contains Snail1 (Snail), Snail2 (Slug) and Snail3 (Smuc). The structure of Snail has two highly conserved domains. The C-terminal zinc finger domains are responsible for binding to the promoter of E-cadherin [26], whereas the N-terminal SNAG domain allows for co-repressors and epigenetic remodeling complex binding [27]. Snail1 is phosphorylated by GSK-β leading to ubiquitination of Snail1 [28]. However, in the presence of Wnt signaling, GSK-β is repressed to stabilize Snail1 activity and trigger EMT [29]. Moreover, Snail1 and Twist1 have independent but collaborative effects on EMT [17]. Snail1 induced cancer stemness via activating the expression of IL-8 or miR-146a [30, 31]. Snail1 is an important mediator in the different signaling pathways that induce EMT, such as the Nijmegen breakage syndrome 1 (NBS1)-Snail1 axis and the TGF-β/Smads/HMGA2/Snail1 axis [32, 33]. In hypoxia-mediated EMT, Notch directly up-regulates Snail1 [34]. NF-κB also up-regulates and stabilizes Snail1 expression via transcriptional and post-translational mechanisms [35]. Similar to Snail1, Snail2 is also phosphorylated by GSK-β and subsequently ubiquitinated by β-Trcp1 [36]. Snail2 is up-regulated by Notch to induce EMT with an increase of cell migration and loss of cell-cell junctions [37]. Snail2 acts synergistically with Snail1 to down-regulate E-cadherin [38]. Snail2 also activates another EMT-TF, ZEB1, and cooperates with it to promote EMT [39]. Regarding anti-apoptotic activity, both Snail2 and Snail1 repress multiple factors involved in programmed cell death [40]. Among these, Snail2 represses p53 expression, leading to apoptosis resistance [41]. Snail3 plays roles in mesodermal formation during embryogenesis but associated studies are limited [42].

The ZEB family, including ZEB1 and ZEB2 (SIP1), contain two zinc-finger clusters to bind to E-boxes and a central region. ZEB1 down-regulates E-cadherin by binding to its promoter with the recruitment of a C-terminal-binding protein (CtBP) and LDS1 to form the CoREST-CtBP corepressor complex for demethylation [43]. Independent of CtBP, chromatin-remodeling protein BRG1 also cooperates with ZEB1 to repress E-cadherin [44]. Alternatively, ZEB2 directly represses tight junction and desmosome proteins, leading to a loss of cell-cell adhesions [45]. ZEB also plays a role in cell invasion [46], loss of polarity and metastasis [47]. The ZEB family is regulated by other EMT-TFs, both Snail1 and Twist1 can up-regulate and cooperate with ZEB1 to induce EMT [48]. TGF-β signaling induces the expression of ZEB1 through Ets-1 [49]. Other growth factors induce ZEB expression through Ras-MAPK and Wnt/β-catenin signaling pathways [14]. In contrast, the ZEB family is repressed by the miR-200 family, which forms a feedback loop to regulate ZEB activity post-transcriptionally [50].

### Inducers for EMT-TFs

TGF-β is a crucial EMT inducer that up-regulates important transcription factors in EMT. Although TGF-β is indicated as a tumor suppressor, cancer cells can disable the tumor-suppressive arm of the TGF-β pathway to promote advanced stage tumor invasiveness and metastasis through EMT [51, 52]. The “TGF-β Paradox” reflects the dynamic alterations that occur within the developing carcinoma and the composition of tumor microenvironment [51, 53]. In cancer cells, TGF-β that deposited in the surrounding stroma induces the expression of ZEB1 and Snail1 in tumor cells, thereby triggering EMT [54, 55]. With Smad complexes and HMGA2, TGF-β also up-regulates the Twist and Snail families and cooperates with them to promote EMT [56]. NF-κB is also indicated essentially for EMT in Ras- and TGF-β–dependent signaling pathways [57]. Furthermore, receptor tyrosine kinases (RTKs) induce EMT through the PI3K-AKT, ERK, MARK or STAT pathway [58] and platelet-derived growth factors induce EMT by promoting nuclear translocation of β-catenin [59]. Vascular endothelial growth factor and Wnt signaling inhibit GSK-3β to stabilize Snail1 and subsequently induce EMT [29, 60]. Hepatocyte growth factor up-regulates Snail1 through the MAPK/Egr-1 signaling pathway [61]. Notch induces the expression of Twist1 and the Snail family through Delta-like or Jagged ligands [62], and HIF-1α can directly regulate Twist1-mediated EMT in response to hypoxia [16]. These signaling pathways participate in crosstalk for intracellular transduction and active the EMT-TFs play critical role in EMT [63].

### EMT-TFs in hematopoiesis

Accumulating evidence demonstrates that EMT-TFs are critical for the regulation of hematopoietic development. Hematopoietic stem cells (HSCs) have the potential to generate various lymphoid and myeloid lineages. Meanwhile, EMT-TFs are critical for regulating hematopoietic development. For example, Dong, C.Y. et al. indicated that Twist1 is highly expressed in HSCs and its expression declines with differentiation, but the enforced Twist1 expression in HSCs increases proliferation, differentiation toward myeloid cells, whereas knockdown of Twist1 impairs the ability [9]. The result indicated.

Twist-1 is not only critical in the maintenance of HSCs and self-renewal capacity but also participated in the development of myeloid lineage [9]. Conversely, Twist2, which markedly increases in mature myeloid populations, negatively regulates myeloid development, through inhibiting RUNX1, C/EBPα, proinflammatory cytokines and promoting regulatory cytokines in myeloid cells [64].

Snail2 is implicated in hematopoietic stem cell self-renewal. The stem cell factor c-kit (SCF/c-kit) signaling pathway, critical in the regulation of hematopoietic stem cell self-renewal [65], up-regulates c-Myc and FoxM1 expression, which promotes Snail2 expression [66]. Moreover, Snail2 negatively regulates c-kit by directly interacting with the c-kit promoter. Thus, the balance of Snail2 and c-kit in the SCF/c-kit-Myc/FoxM1/Snail2 pathway controls hematopoietic stem cell self-renewal [67].The Snail family is also involved in lymphoid development. Initially, the E2A-HLF fusion gene was found to transform pro-B lymphocytes by blocking apoptosis [12] and Inukai et al. further discovered that Snail2 was up-regulated by the E2A-HLF oncoprotein in a pre-B cell line [68]. Furthermore, the expression of Snail2 prolonged IL-3 dependent cell survival in the absence of cytokines and promoted pro-B cell malignant transformation, which demonstrates the anti-apoptotic activity of Snail2 in lymphoid development. In contrast, Dahlem et al. found that overexpression of Snail3 suppressed lymphoid cell differentiation [69]. A Snail3-expression retrovirus vector was transduced into irradiated mice, which resulted in normal numbers of hematopoietic precursor cells but impairment of lymphocyte development. The same study also indicated that Snail3 was overexpressed in developing T cells and knockdown of Snail3 alone had no effect on T cell development [69]. These data suggested that Snail2 played a more important role in lymphoid development.

Moreover, ZEB2 is essential for murine embryonic hematopoietic differentiation and mobilization [70]. ZEB1, but not ZEB2, which is highly expressed in T cells from the thymus [71], contains repressor domains that function in T lymphocytes [72].

### EMT-TFs in myeloid malignancies

**Fig. 1:**
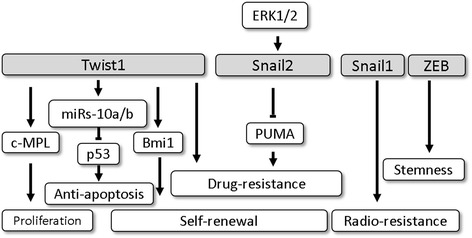
The role of epithelial-mesenchymal transition-transcription factors in myeloid malignancies. The major EMT-TFs in myeloid malignancies are Twist1, Snail1, Snail2, and ZEB. Twist1 up-regulates proto-oncogene c-MPL expression to promote leukemia cell growth. The Twist1/miRs-10a/b axis down-regulates p53-mediated cell apoptosis. Twist1 up-regulates and cooperates with Bmi1 to maintain leukemia cell self-renewal. Twist1 expression is also associated with drug resistance to daunorubicin and mitoxantrone in AML and imatinib mesylate in CML. ERK1/2-induced Snail2 expression leads to a down-regulation of PUMA and enhanced drug resistance in AML and CML. Alternatively, overexpression of Snail1 results in anti-apoptotic activity and radio-resistance. ZEB2 is an essential transcription factor for AML cells to maintain stemness

These major EMT-TFs that play a role in myeloid malignancies are summarized in Fig. 1. Twist1 is highly expressed in human leukemia stem cells, which maintain leukemia stem cell function [9, 73]. Twist1 promotes leukemia cell growth and colony formation through the Twist/c-MPL axis, and knockdown of Twist1 inhibits tumor growth [73]. Twist1 also indicated significantly increases in CD34+ myelodysplastic syndrome (MDS) cells by regulating the anti-apoptotic activity through the Twist1/ miRs-10a/b/p53 axis, therefore, the expression of Twsit1 is and associated with disease progression [74, 75]. Bmi1, which maintains chromatin silencing through repression of the INK4A–ARF locus [76], is essential in Twist1-induced EMT [10]. Park IL et al. demonstrated that Bmi1 was essential for self-renewal in HSCs and a lack of Bmi1 resulted in marked reduction in HSCs [77]. In leukemia stem cells, Lessard J et al. found that Bmi1 had a key role in regulating the proliferative activity [78], and thus its expression was associated with the progression from MDS to acute myeloid leukemia (AML) [79]. In contrast, repression of Bmi1 impaired self-renewal and induced apoptosis in leukemia stem cells [80]. Recently, evidence has indicated that Twist1 confers AML cell self-renewal and apoptosis resistance via a Twist1-Bmi1 axis [11]. Taken together, these studies highlight the importance of the Twist1-Bmi1 axis in the regulation of self-renewal in both hematopoietic and leukemia stem cells.

The Twist1 expression may also cause drug resistance. Nan et al. reported that Twist1 expression was associated with the resistance to daunorubicin and mitoxantrone, which leads to the poor prognosis. However, the molecular and cytogenetic risk was unknown in this study [73]. On the contrary, another study showed that the aggressive disease phenotype of AML with overexpression of Twist1 had a higher response to cytarabine treatment, leading to a favorable outcome [11]. This result indicated that Twist1 overexpression was associated with good molecular/cytogenetic risk, which may explain the better prognosis. On the other hands, the interaction between Twist1 and signaling pathways in a low-risk AML subtype requires further study. Regarding chronic myelogenous leukemia (CML), the Twist1 expression level in CD34+ CML cells acted as a prognostic factor and also a biomarker for early detection of tyrosine kinase inhibitor resistance irrespective of any other resistance mechanism. Although the link between Twist1 and Bcr-Abl translocation is unclear [81], Twist1 may still serve as an important predictive marker for treatment and prognosis in myeloid malignancies. Twist2, in contrast to Twist1, was identified as a negative regulator of myeloid progenitor cells [64]. Twist2 directly up-regulated p21 expression, which inhibited the growth of AML cells [82]. The frequent hypermethylation of Twist2 in human AML resulted in the inactivation of Twist2 and subsequent tumor growth.

In Bcr-Abl^p190^ transgenic mice, Pe’rez-Mancera et al. found that Snail2 was up-regulated and essential for promoting leukemogenesis [83]. Snail2 expression also conferred leukemia cell resistance to apoptosis, whereas blocking Snail2 induced apoptosis [84]. The expression of Snail2 is induced by ERK1/2, which leads to down-regulation of p53 up-regulated modulator of apoptosis (PUMA) and subsequent chemoresistance to cytarabine [85] and Adriamycin [86]. In a CML cell line, Mancini et al. showed that Bcr-Abl^p210^ regulated Snail2 expression via ERK1/2, which led to imatinib mesylate resistance [87]. In Snail1-expressing mice, leukemia formation could be induced and the expression of Snail1 also promoted resistance to programmed cell death resulting in radio-resistance [88]. Taken together, the Snail family, especially Snail2, may control self-renewal, anti-apoptosis and treatment resistance in leukemia, which makes this family a potential target for leukemia treatment. Furthermore, ZEB2 was identified as an essential transcription factor for leukemia cell stemness maintenance, and loss of ZEB2 led to aberrant differentiation and decreased proliferation [89].

### EMT-TFs in lymphoid malignancies

**Fig. 2:**
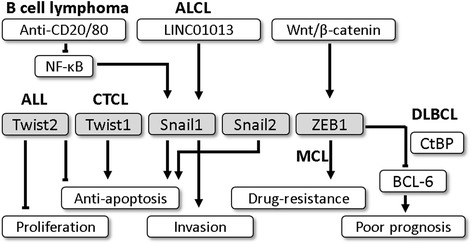
The role of epithelial-mesenchymal transition-transcription factors in lymphoid malignancies, In lymphoid malignancies, Twist1 is overexpressed in Sezary syndrome (SS), a rare cutaneous T cell lymphoma (CTCL), and its expression may be associated with anti-apoptotic effects. In contrast, the expression of Twist2 inhibits tumor growth and induces apoptosis in acute lymphoblastic leukemia (ALL). In B cell lymphoma, NF-κB and the upregulation of Snail1 promote lymphoma cell resistance to apoptosis, whereas anti-CD20 and anti-CD80 monoclonal antibodies reverse the effect. Moreover, non-coding RNA LINC01013 activates Snail1 and enhances the invasion ability of anaplastic large-cell lymphoma (ALCL). Snail2 has anti-apoptotic activity during lymphoid development. In diffuse large B cell lymphoma (DLBCL), the Wnt/β-catenin signaling pathway induces the expression of ZEB1, which cooperates with CtBP to inhibit Bcl-6, leading to the poor prognosis of B cell lymphoma. Similarly, Wnt signaling induces ZEB1 expression in a mantle cell lymphoma cell line and makes MCL cells resistant to chemotherapy

The roles of EMT-TFs in lymphoid malignancies are summarized in Fig. 2. In acute lymphoblastic leukemia (ALL), Thathia et al. found that inactivation of Twist2 via promoter hypermethylation was common in humans [90]. Re-introduced Twist2 gene expression in an ALL cell line inhibited tumor growth and induced apoptosis to cytotoxic agents. Hence, the expression of Twist2 is a negative regulator of ALL cell survival, which is consistent with its role in AML as mentioned above [82]. These data identify Twist2 as a potential target for drug development for acute leukemia.

In T cell lymphoma, Sezary syndrome (SS) is a rare cutaneous T cell lymphoma (CTCL) with aggressive characteristics, whereas mycosis fungoides (MF) is the most common type of CTCL with an indolent course. By identifying more than 100 genes, Doorn et al. found significantly increased Twist1 gene expression in CD4+ T cells from the peripheral blood of SS patients compared with that from skin lesions of MF patients [91]. Using qPCR for validation, Twist1 mRNA was consistently up-regulated in SS patients but almost undetectable in control samples. Vermeer et al. demonstrated that part of Twist1 overexpression was due to the gain of a chromosomal region that harbors the Twist1 gene [92]. Other mechanisms of Twist1 overexpression in SS have also been reported. For instance, Wong et al. found the genome-wide and Twist1 promoter hypomethylation led to the aberrant expression of Twist1 in SS [93]. Goswami et al. further demonstrated that the frequency and intensity of Twist1 expression by IHC were correlated to clinical stages of SS. The co-expression of Twist1, c-Myc, and p53 suggest that the role of Twist1 in SS progression may be associated with anti-apoptotic activity [94]. With accumulating evidence demonstrating the importance of Twist1 in SS, Michael L. suggested expressing a combination of four genes, including Twist1, to make early, reliable diagnoses of SS [95]. Taken together, these data addressed the critical role of Twist1 in SS. The value of Twist1 in pathogenesis, diagnosis and therapeutic target needs further investigation. Alternatively, Twist2 is involved in Sezary cell apoptosis. Koh et al. found that Twist2 directly repressed the activity of the CD7 promoter via chromatin acetylation, which resulted in resistance to galectin-1-induced apoptosis [96]. Limited data have been reported on EMT-TFs in other types of T cell lymphoma. In anaplastic large-cell lymphoma (ALCL), Chung et al. found that a long non-coding RNA, LINC01013, activated Snail1 and downstream fibronectin to enhance the invasion of an ALCL cell line [97]. In diffuse large B cell lymphoma (DLBCL), Lemma et al. indicated that ZEB1 and Slug expression correlated with adverse disease presentation, and the nuclear expression of ZEB1 seems to be the major one that associated with unfavorable outcomes [98]. The previous study showed that ZEB1 and CtBP formed a complex to repress the promoter of B-cell lymphoma 6 protein (Bcl-6), a proto-oncogene that serves as a good prognostic factor in DLBCL [99]. This finding may explain the poor prognosis of ZEB1 expression in DLBCL.

ZEB1 also serves as a poor prognostic factor in mantle cell lymphoma (MCL) [100, 101]. Sanchez-Tillo et al. demonstrated that ZEB1 expression in MCL cells was induced by Wnt signaling pathway, and the Chromatin immunoprecipitation (ChIP) assay further confirmed that Beta-catenin binds to the promoter of ZEB1 to regulate its expression [101]. The activation of ZEB1 regulates genes involved in proliferation,apoptosis, and determines a differential response to chemotherapy drugs [101]. These data suggest that ZEB1 is a potential biomarker for prognosis and a therapeutic target for MCL [101]. In B cell lymphoma, anti-CD20 is widely used to treat CD20+ B cell lymphoma through antibody-dependent cellular cytotoxicity (ADCC) and sensitization to chemotherapy or TRAIL-mediated apoptosis [102]. Furthermore, Baritaki et al. found anti-CD20 inhibited NF-κB activity and subsequent Snail1 down-regulation, which led to PTEN expression and AKT inactivation [103]. After transfection with Snail1 siRNA, cells were resistant to TRAIL-induced apoptosis. However, knockdown of Snail1 showed opposite results. Similarly, Snail1 also plays a role in B cell lymphoma with anti-CD80 monoclonal antibody treatment [104]. CD80 is a member of B7 co-stimulators expressed on the surface of T cells and activated B cells. Martinez-Paniagua et al. found that anti-CD80 down-regulated NF-κB and downstream protein expression, which suppressed the expression of Snail1 and YY1, leading to the increase of cisplatin- and TRAIL-induced apoptosis of CD80+ Burkitt’s B-NHL cell lines. Therefore, Snail1 may be a novel target for enhancing the response of monoclonal antibodies and cytotoxic agents.

## Conclusions

EMT has received increasing attention in hematological malignancies. As in solid tumors, EMT-TFs are also implicated in cancer proliferation, stemness, anti-apoptosis and drug resistance in hematological malignancies. Notably, evidence shows that EMT-TFs are related to chemotherapy and radiotherapy resistance in both myeloid and lymphoid malignancies. These findings make them potential targets for combination with conventional treatments. Determining the diagnostic and prognostic value of EMT-TFs is worthy of future study.

## Abbreviations

ADCC: Antibody-dependent cellular cytotoxicity
ALL: ac Ute lymphoblastic leukemia
AML: Acute myeloid leukemia
BCL-6: B-cell lymphoma 6 protein
Bmi1: B cell-specific Moloney murine leukemia virus integration site 1
ChIP: Chromatin immunoprecipitation
CML: Chronic myelogenous leukemia
CTCL: Cutaneous T cell lymphoma
DLBCL: Diffuse large B cell lymphoma
EMT: Epithelial-mesenchymal transition
EMT-TFs: epithelial-mesenchymal transition-transcription factors
GSK-β: Glycogen synthase kinas-β
HSCs: Hematopoietic stem cells
MAPK: Mitogen-activated protein kinase
MCL: Mantle cell lymphoma
MDS: Myelodysplastic syndromes
MET: Mesenchymal-epithelial transition
MF: Mycosis fungoides
NBS1: Nijmegen breakage syndrome 1
NF-κB: Nuclear factor kappa-light-chain-enhancer of activated B cells
PUMA: p53 up-regulated modulator of apoptosis
RTKs: Receptors tyrosine kinases
SCF/c-kit: stem cell factor/c-kit signaling pathway
Snail1: Snail family zinc finger 1
SS: Sezary syndrome
TGF-β: Transforming growth factor-β
TNF-α: Tumor necrosis factor-α
Twist1: Twist family bHLH transcription factor 1
ZEB1: Zinc finger E–box binding homeobox 1
ZEB2: Zinc finger E–box binding homeobox 2
